# Editorial: Tumor microenvironment in bladder cancer

**DOI:** 10.3389/fonc.2023.1208196

**Published:** 2023-05-03

**Authors:** Sergei Kusmartsev, Ja Hyeon Ku, Fabio Grizzi

**Affiliations:** ^1^ Department of Urology, University of Florida, Gainesville, FL, United States; ^2^ Department of Urology, Seoul National University Hospital, Seoul, Republic of Korea; ^3^ Department of Immunology and Inflammation, IRCCS Humanitas Research Hospital, Rozzano, Milan, Italy; ^4^ Department of Biomedical Sciences, Humanitas University, Pieve Emanuele, Milan, Italy

**Keywords:** bladder cancer, tumor microenvironment (TME), cancer inflammation, tumor heterogeneity, cancer immunotherapy

Bladder cancer is one of the most common cancers worldwide and one of the most prevalent genitourinary cancers, particularly in men. Bladder cancer is a heterogeneous disease classified into major two sub-types: non-muscle invasive bladder cancer (NMIBC) which accounts for approximately 75% of all bladder cancer cases and the remaining 25% have muscle-invasive bladder cancer (MIBC) (1). Clinically, NMIBCs are typically classified as tumors that have a high likelihood of recurring but a low tendency to progress, leading to a favorable survival rate after standardized therapy. This group encompasses cases of urothelial carcinoma *in situ* as well as Ta and T1 cases with low-to-high-grade. In contrast, MIBCs comprises T2-T4 disease. Importantly, the proportion of patients diagnosed with bladder cancer, MIBC carries a significant risk of death that has not significantly changed in decades. For high-risk NMIBC cases after transurethral resection, the standard of care involves adjuvant intravesical Bacillus Calmette-Guérin (BCG) immunotherapy. In MIBC, neoadjuvant chemotherapy followed by radical cystectomy is the preferred treatment option. In cases of locally advanced or metastatic MIBC, treatment may also involve biomarker-guided immune checkpoint inhibitors (ICI), targeted therapies, or other novel drug conjugates (2). Although there has been significant progress in bladder cancer research, as well as in the optimization of therapeutic approaches, the survival rate and prognosis remain poor, particularly for patients with advanced bladder cancer. Furthermore, once bladder cancer metastasizes, it becomes one of the leading causes of genitourinary cancer-related mortality, with the average life expectancy being less than two years. Therefore, to deepen our understanding of molecular and cellular mechanisms of bladder cancer progression and recurrence, develop novel therapeutic approaches including the identification of novel molecular targets and improving the efficacy of the existing therapeutic modalities (3, 4).

The tumor microenvironment in bladder cancer is highly complex (**Figure 1**). Bladder cancer is characterized by a highly immunosuppressive microenvironment with frequently up-regulated expression of inhibitory ligand PD-L1 and the strong presence of myeloid-derived suppressor cells and tumor-associated macrophages (5, 6). The tumor-associated immune suppression represents a major obstacle to successful cancer immunotherapy, particularly in patients with advanced and metastatic bladder cancer.

**Figure 1:**
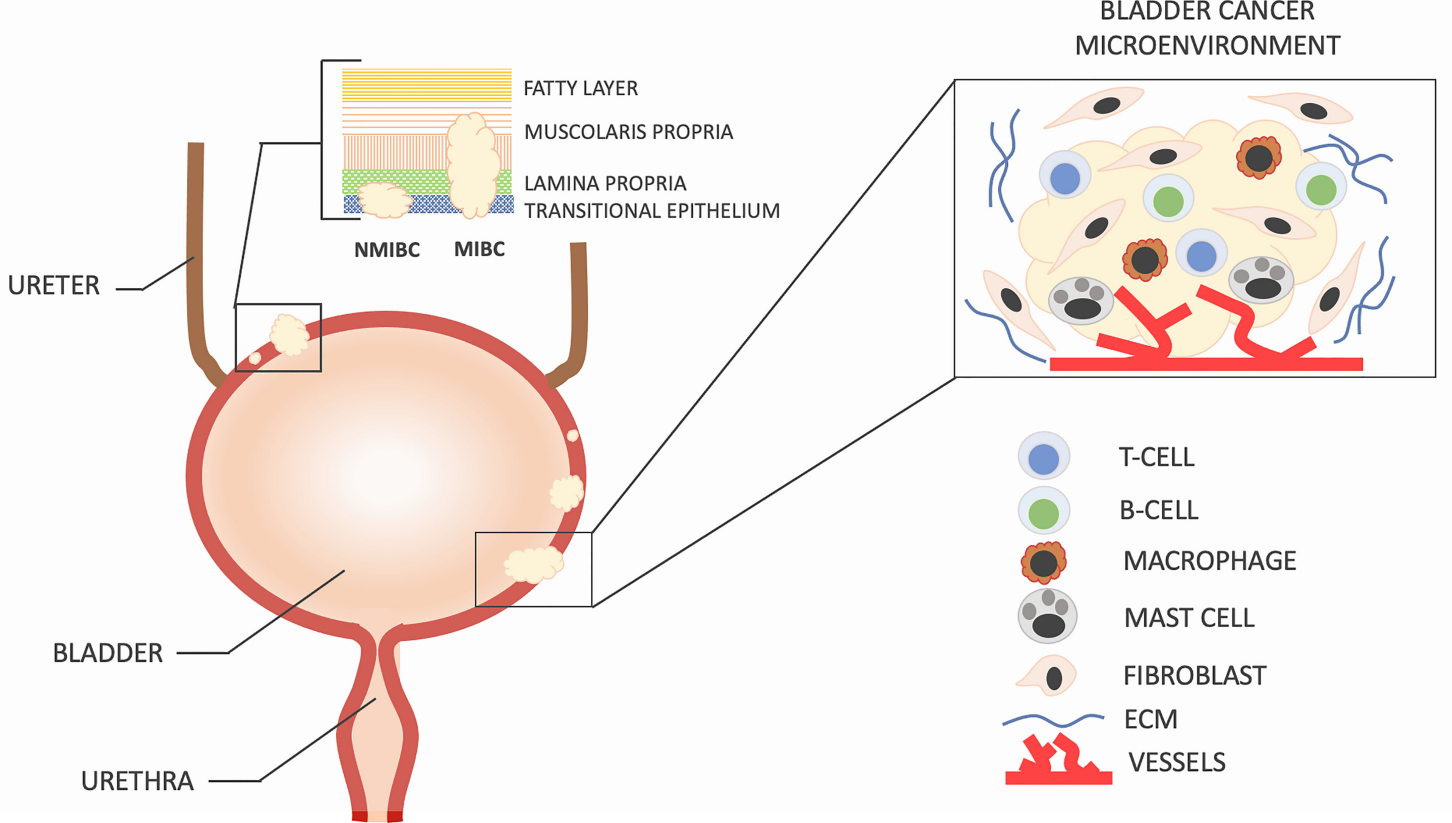
Bladder cancer can be classified into two groups based on the extent of tumor invasion. Tumors that penetrate the detrusor muscle are referred to as muscle-invasive bladder cancer (MIBC) and have a higher likelihood of metastasis to other organs and lymph nodes. On the other hand, non-muscle-invasive bladder cancer (NMIBC) is found in approximately 75% of newly diagnosed patients, while the remaining 25% have MIBC or metastatic disease. The development and recurrence of bladder cancer are not solely determined by the molecular characteristics of cancer cells but also by the intricate interactions between these cells and the surrounding microenvironment, which is composed of specialized cells such as immune cells, activated fibroblasts, endothelial cells, extracellular matrix, and secreted soluble factors. Changes in cell density, type, and spatial arrangement within the microenvironment are responsible for the nonlinear progression and recurrence of bladder cancer.

The tumour-associated immune suppression is supported by cancer inflammation through elevated production of chemokines and cytokines, which promotes constant mobilization of tumor-supporting cells such as immunosuppressive myeloid cell subsets, precursors of endothelial cells, and cancer-associated fibroblasts (7, 8). Cancer-related inflammation in bladder cancer is mediated by enhanced degradation of tumor-associated hyaluronan as well as by secretion of eicosanoids, including prostaglandins and leukotrienes. The importance of the prostanoid and leukotriene biosynthetic pathways in carcinogenesis and chronic inflammation is supported by clinical trials, animal experiments, and epidemiological studies (9–13).

Stromal cells, such as cancer-associated fibroblasts (CAFs) play an important role in the progression of bladder cancer with significant implications on tumor cell signaling, epithelial to mesenchymal transition (EMT), and the capacity of the modulate immune system (Burley et al.). Both, CAFs and tumor-associated macrophages are involved in the formation of an immunosuppressive tumor microenvironment by interacting with the cancer epithelial cells and creating a milieu that renders activated infiltrating T-lymphocytes to apoptosis or anergy (14, 15).

Despite the recent significant progress in cancer research, the precise mechanisms of bladder cancer progression and resistance to cancer therapy are still unclear and need further elucidation. This Research Topic, therefore, provides a great opportunity to highlight and promote research in this area. It is a concise collection of studies that illustrates the recent findings in bladder cancer research.

Bladder cancer is inherently pro-inflammatory, and increased production of various chemokines, cytokines, and growth-factors is thought to be critical for tumor development, maintenance, and progression. One such chemokine is CXCL12 (SDF-1), which together with its cognate receptor CXCR4 is reported to be elevated in various tumors, and distant metastases, which correlates with poor survival in cancer patients. Liu et al. demonstrate that increased expression of CXCL12 in bladder cancer was associated with B-lymphocytes, mast cells, tumor-associated macrophages, and several subsets of T-lymphocytes and dendritic cells.

Research by Wang et al. is focused on PSMD2 (26S proteasome non-ATPase regulatory subunit 2), which is an enzyme playing a critical role in maintaining the homeostasis of the cellular proteome. PSMD2 expression was found to be significantly elevated in bladder cancer tissue as compared to non-malignant bladder tissue. High PSMD2 expression correlated with the cell cycle, antigen processing and presentation, JAK-STAT signaling pathway, TLR signaling pathway, and P53 and MAPK signaling pathway. Knockdown of PSMD2 could remarkably inhibit wound healing and colony formation efficiency by cancer cells. Additional analysis revealed that overexpressed PSMD2 is positively related to the Th2 cell infiltrates and immune escape markers, and negatively associated with the infiltrating levels of NK T cells and CD8^+^ T lymphocytes.

The study by Wu et al. employs a wide variety of techniques to study pyrimidine metabolism in the bladder tumor microenvironment. The authors demonstrate that several genes involved in the regulation of pyrimidine are up-regulated in bladder cancer tissue as compared to natural bladder tissue. More specifically, these genes were found to be overexpressed in the high-risk sample in the absence of other clinical symptoms, showing that they can predict the outcome of bladder cancer. The prognostic model, copy number variations (CNVs), single nucleotide polymorphism (SNP), and drug sensitivity all showed substantial connections between those genes.

The work by Li et al. is focused on immune and stromal components of tumor microenvironment in bladder cancer. They show that the proportion of immune and stromal components within the cancer tissue was associated with the prognosis of bladder cancer. Based on the scores of immune and stromal components, authors demonstrate that higher CD8^+^ T lymphocytes, higher tumor mutational burden, and higher chemosensitivity were found in the low-risk group, which presented a better prognosis.

The study by Chen et al. use a single-cell RNA sequencing and bioinformatics to compare MIBC, NMIBC, and non-pathologic adjacent bladder tissues. Using weighted gene co-expression network analysis (WGCNA), the protein–protein interaction (PPI) network mutual analysis, and the Kaplan–Meier survival prognosis analysis, authors show that six key genes were associated with the prognosis of bladder cancer: VEGFA, ANXA1, HSP90B1, PSMA7, PRDX6, and PPP1CB. The dynamic change of the expression distribution of six genes on the pseudo-time axis was further verified by cell pseudo-time analysis.

Xiao et al., using bioinformatics and EMT-related gene patterns, created two EMT-focused clusters. The authors provide evidence indicating that cluster #2 exhibits an inflammatory tumor microenvironment phenotype and is linked to a poorer prognosis in cancer patients compared to cluster #1.They also developed and validated an EMT-based risk score that includes 7 candidate genes: ANXA10, CNTN1, FAM180A, FN1, IGFL2, KANK4, and TOX3. Patients with high EMT risk scores had lower overall survival with high predictive accuracy both in the training cohort and validation cohort.

The study by Shen et al. is focused on circular RNA (circRNAs). This work provides a circRNA-associated gene model which may have a potential value for the clinical prognosis, immunotherapeutic responsiveness, and chemotherapeutic sensitivity in patients with bladder cancer. Notably, the model was validated using several external datasets including GSE13507, GSE31684, GSE48075, IMvigor210, and GSE32894.

Overall, the knowledge offered in these studies could provide a clearer picture of the complex TME landscape in bladder cancer. However, much more extensive studies are still required for a better understanding of TME and mechanisms of therapeutic resistance with the ultimate goal to boost therapeutic efficacy and improve clinical outcomes.

## Author contributions

All authors listed have made a substantial, direct, and intellectual contribution to the work, and approved it for publication.
